# Acute Congestion of the Kidneys with Posterior Paralysis

**Published:** 1881-01

**Authors:** J. Cuff


### III.—A CASE OF ACUTE CONGESTION OF THE KIDNEY IN A GRAY GELDING, COMPLICATED WITH PARALYSIS IN THE HIND EXTREMITIES.

---

DEAR SIR—Thinking that the following case may prove interesting to many of the readers of the JOURNAL, I am induced to forward it for insertion. The subject of this communication is a powerful gray gelding, five years old, and about fifteen cwt. (1500), the property of Matthew White, Esq., malster, foot of Bethune street, this city.

*History.*—On the morning of February 27th, 1880, at 7 A. M., the horse left stable in Bethune street to all appearances in the best of health; when he got as far as Market street he lost all power in posterior extremities, and went down. The driver and others used all means at their command to raise him, but to no purpose. About 9:30 A. M., same day, at the request of owner, I was called. When I arrived at Market street, found my case lying on left side, unable to get up. I called a few assistants, had him turned over on right side, but with no better results. The weather being frosty, with some snow on the ground, I ordered blankets and straw placed around him, and alcoholic stimulants in the shape of Irish whiskey one pint, and water eight ounces. I next sent for Mr. H. Bergh's van, which arrived about 2 P. M. I must here commend and thank the ambulance aids for their prompt appearance and their careful manner in handling the poor dumb creature. On arriving at my Infirmary, had horse removed from ambulance and placed in a large, well-ventilated box-stall, 10 x 12 feet; gave alcoholic stimulants freely, followed with

| | |
|---|---|
| Linseed oil, | 1 pint, |
| Barbadoes aloes, | 3 drachms, |
| Pulv. ginger, | 2 drachms. |

Used friction and liniments over loins and extremities; gave nux vomica in powder 2 drachms, ginger and gentian a. a. 2 drachms in bolus. Next had my patient placed in slings, then gave enema—soap-suds six quarts, oil of turpentine 2 ounces. Next, by the means of catheter, removed about two quarts of dark-colored urine, tinged with blood and slightly albuminous. 6 P. M., my patient very weak; pulse quick and small, almost imperceptible. Gave

| | |
|---|---|
| Extract digitalis, . . . . . . | 15 grains, |
| Carbonate ammonia, . . . . . . | 2 drachms, |
| Cinchona, in powder, . . . . . . | 1 drachm, |
| Ginger, in powder, . . . . . . . | 1 drachm, |

in bolus; left linseed tea for drink, and gave four quarts of bran mash; ordered flannel bandages on all fore extremities, and left him in charge of night watchman. February 28th, at 7 A. M., found patient much better; pulse 45 per minute, extremities warm, patient somewhat firmer on his limbs. Gave nux vomica, in powder, 2 drachms; sulphate of iron half drachm, in bolus, every six hours, followed with whiskey four ounces and water 4 ounces, twice a day.

February 29th.—Same treatment continued. Gave oatmeal gruel in bran mash. March 1st, 8:30 A. M., pulse normal, but bowels very active. Discontinued the use of linseed tea; wheaten flour, half-pound in a pail of chilled water, substituted.

Treatment about the same until March 5th. Patient continuing to improve, I stopped the use of nux vomica, and gave in place of it—

| | |
|---|---|
| R.—Extract digitalis, . . . . . . | 10 grains, |
| Sulphate iron, . . . . . . . | ½ drachm, |
| Iodidi potassii, . . . . . . . . | ½ drachm, |
| Gentian et ginger, . . . . . . . | a. a. ½ drachm, |

in bolus, one daily for five days. His diet consisted of ground malt and bran mash, with hay sparingly.

March 15th.—Patient doing well. Removed him from slings in daytime, but had him put back at night. Continued the use of tonics and stimulants each daily, till March 25th, when I removed my patient from slings for good. He was then able to get up and down himself. All medical agents were suspended; gave nutritious diet, walking exercise, etc., etc.

April 2d.—Patient discharged. Went to work about April 10th, and has worked and done well ever since. J. CUFF, D. V. S.

